# 

**Published:** 1888-03

**Authors:** 


					﻿Vol. IX.
MARCH. 1888.
No. 3.
THE
iwi twnipraen wr:
A /V\ OlNWWTf J. nire wr B T
DEVOTED TO
DENTAL AND ORAL SCIENCE.
W. C. BARRETT, M. D., D. D. S.,
EDITOR.
The New York Dental Journal Association,
PUBLISHERS,
No, 35 West <L€>tli Street, New York.
AND
No. 208 Franklin St., Buffalo, N.Y.
$2.50 Per Annum in Advance, .... Single Copies, 25 Cents.
Entered at the Post-Office, Buffalo, N. Y., as Second-Class matter.
				

